# Differentiation of Bone Mesenchymal Stem Cells Into Vascular Endothelial Cell-Like Cells Using Functionalized Single-Walled Carbon Nanotubes

**DOI:** 10.3389/fbioe.2022.913080

**Published:** 2022-06-07

**Authors:** Feng Luo, Ruyi Li, Huaping Zheng, Yichen Xu, Linxin Yang, Changxing Qu, Guang Hong, Qianbing Wan

**Affiliations:** ^1^ State Key Laboratory of Oral Diseases, National Clinical Research Center for Oral Diseases, West China School of Stomatology, Sichuan University, Chengdu, China; ^2^ State Key Laboratory of Biotherapy and Cancer Center, West China Hospital, West China Medical School, Sichuan University and Collaborative Innovation Center for Biotherapy, Chengdu, China; ^3^ Liaison Center for Innovative Dentistry, Graduate School of Dentistry, Tohoku University, Sendai, Japan; ^4^ Department of Prosthetic Dentistry, Faculty of Dental Medicine, Airlangga University, Surabaya, Indonesia

**Keywords:** vascularization, bone regeneration, single-walled carbon nanotubes, angiogenesis, vascular endothelial cell-like cells

## Abstract

Carbon nanotubes (CNTs) are a promising bioactive scaffold for bone regeneration because of their superior mechanical and biological properties. Vascularization is crucial in bone tissue engineering, and insufficient vascularization is a long-standing problem in tissue-engineered scaffolds. However, the effect of CNTs on vascularization is still minimal. In the current study, pristine single-walled carbon nanotubes (SWNTs) were purified to prepare different ratios of SWNTs/EDC composites, and their surface morphology and physicochemical properties of SWNTs/EDC were studied. Furthermore, the effect of SWNTs/EDC on vascularization was investigated by inducing the differentiation of bone mesenchymal stem cells (BMSCs) into vascular endothelial cell-like cells (VEC-like cells). Results showed that SWNTs/EDC composite was successfully prepared, and EDC was embedded in the SWNTs matrix and uniformly distributed throughout the composites. The AFM, FTIR spectra, and Raman results confirmed the formation of SWNTs/EDC composites. Besides, the surface topography of the SWNTs/EDC composites presents a rough surface, which may positively affect cell function. *In vitro* cell culture revealed that SWNTs and SWNTs/EDC composites exhibited excellent biocompatibility and bioactivity. The SWNTs/EDC composite at mass/volume ratios 1:10 had the best enhancement of proliferation and differentiation of BMSCs. Moreover, after culture with SWNTs/EDC composite, approximately 78.3% ± 4.2% of cultured cells are double-positive for FITC-UEA-1 and DiI-Ac-LDL double staining. Additionally, the RNA expression of representative endothelial cell markers VEGF, VEGF-R2, CD31, and vWF in the SWNTs/EDC composite group was significantly higher than those in the control and SWNTs group. With the limitation of our study, the results suggested that SWNTs/EDC composite can promote BMSCs differentiation into VEC-like cells and positively affect angiogenesis and bone regeneration.

## 1 Introduction

Many people currently suffer from bone loss due to trauma or disease. Bone tissue engineering aims to develop strategies to treat bone loss to overcome the disadvantages of current clinical autograft and allograft treatments (Hu et al., 2019). Recently, carbon nanotubes (CNTs) have drawn considerable attention due to their unique physicochemical and mechanical properties, their ability to enhance cell functionality and direct differentiation, and their desirable framework for cellular proliferation and survival (Pei et al., 2019). CNTs, including multi-walled carbon nanotubes (MWNTs) and single-walled carbon nanotubes (SWNTs), have increasingly been used as bone scaffolds in tissue engineering (Tohidlou et al., 2019; Liu et al., 2020). As reported, the diameters of SWNTs are generally within the range of 1–2 nm, and the theoretical surface area of SWNTs is 1,315 m^2^/g, lower than MWNTs’ 100 m^2^/g, which makes it a potential candidate for biomedical applications (Arumugam and Ju, 2021). Previous studies demonstrated that by incorporating SWNTs, SWNTs/HA composite coatings improved cell adhesion, proliferation, and alkaline phosphatase (ALP) activity compared to pure HA coatings and pure Ti (Pei et al., 2014). Furthermore, we fabricated CNT-based scaffolds and investigated their properties *in vitro* and *in vivo* (Luo et al., 2017). The *in vitro* and *in vivo* results suggest that CNT-based scaffolds enhance osteoblast differentiation and promote the formation of new bone, resulting in greater density and bone regeneration. Interestingly, a small amount of carbon nanotube doping had little effect on cytotoxicity (Li et al., 2020). CNTs also displayed minimal local inflammation and were well-integrated into the newly formed bone after bone regeneration (Costa et al., 2013).

Bone tissue is highly vascularized and essential in transporting nutrients, oxygen, and growth factors during bone regeneration. Insufficient vascularization is a long-standing problem in tissue-engineered scaffolds (Luo et al., 2022). A graft combining osteogenic and angiogenic cells will enhance new bone formation. CNTs can be considered osteoproductive materials in bone regeneration because they can promote the adhesion and proliferation of osteoblasts on the material surface (Islam et al., 2020). Therefore, as a promising scaffold biomaterial in bone regeneration, it should also be crucial to determine the effect CNTs on vascularization. Duan et al. reported that incorporating CNTs into poly(L-lactide) nanofibrous scaffolds can effectively promote bone regeneration and enhance vascularization (Duan et al., 2015). Besides, Kumar et al. found that N2-functionalized carbon nanomaterials are highly efficient at providing cell-anchorage to endothelial cells and can promote pro-angiogenic factors in cells. These studies suggest that CNTs may play an essential role in angiogenesis and vascularization (Kumar et al., 2019). These might explain the superiority of carbon nanotubes in bone regeneration and provide a theoretical and experimental basis for its application in bone tissue engineering. However, research on CNTs in vascularization is still minimal.

Bone mesenchymal stem cells (BMSCs) are a promising candidate for bone tissue engineering. BMSCs can differentiate into a variety of cells, including vascular endothelial cells (VECs), osteoblast, osteoclasts, and so on (Chen et al., 2018). VECs are an essential component of capillaries, providing blood supply and excretion of waste products from tissues, which play a central role in angiogenesis. Furthermore, VECs can promote vascularization and, indirectly, new bone formation during bone healing. To promote vascularization and improve bone regeneration, many researchers are focused on finding biomaterials that can induce the differentiation of BMSCs into VECs (Su et al., 2010; Chen et al., 2018). Thus, we can evaluate the effect of functionalized SWNTs on vascularization by inducing BMSCs differentiation into VECs with SWNTs.

In the present study, pristine SWNTs are purified and functionalized with 1-ethyl-3-(3-dimethyl aminopropyl) carbodiimide (EDC) to prepare SWNTs/EDC composites. BMSCs were used to explore further the ability of SWNTs/EDC composites to promote differentiation into VEC-like cells. We characterize SWNTs/EDC composites physically, chemically, and morphologically and study the *in vitro* responses of BMSCs to these composites regarding adhesion, proliferation, and differentiation of VEC-like cells. Then the optimal ratio of SWNTs to EDC was determined, and the effect of SWNTs on vascularization was explored. The null hypothesis is that SWNTs/EDC composites can promote BMSCs differentiation into VEC-like cells and positively affect angiogenesis and bone regeneration.

## 2 Materials and Methods

### 2.1 Materials

Single wall carbon nanotubes functionalized with carboxylate groups (SWNTs-COOH; 1–2 nm in diameter, 5–30 μm in length, -COOH content 2.73 wt%, purity >90 wt%) were Chengdu Organic Chemicals Co., Ltd. (Chengdu, China). EDC was obtained from Sigma-Aldrich (St Louis, MO). The other chemicals were analytical grade and used without further purification.

### 2.2 Preparation of Functionalized SWNTs, SWNTs/EDC

In the preceding process, pristine SWNTs (Group A: 20 mg, Group B: 40 mg, Group C: 60 mg, and Group D: 80 mg) were purified by sintering in a vacuum furnace at 400°C at a heating rate of 30°C/min for 1 h. The weight loss of the prepared samples was evaluated. After cooling to room temperature, 10 mg of purified SWNTs were added to pure alcohol at mass/volume ratios of 1:1, 1:5, and 1:10. We stirred the mixtures with a magnetic stirrer for 6 h and sonicated them (200 w) for 0.5 h with an ultrasonic cleaner for VGT 2300B (Weigute, Shenzhen, China) to obtain homogeneous and stable suspensions. The suspensions were centrifuged at 10000 rpm for 5 min using a TDL-40B centrifuge from Anke (Anting, Shanghai, China) to remove undispersed SWNTS bundles. Afterward, EDC was dissolved in pure water according to its solubility and then mixed with SWNTs suspensions at a volume ratio of 1:1 at room temperature for 2 h. The pH value of these suspensions was maintained at 4.75 using hydrochloric acid (HCl, 0.01 mg/L), and these supernatant suspensions were used for the following experiments.

### 2.3 Characterization of Materials

Briefly, pristine SWNTs, purified SWNTs, EDC, and different ratios of SWNTs/EDC composite suspensions were dropwise added onto glass slides and then air-dried. Scanning electron microscopy (SEM) was used to examine the surface morphology of these specimens. AFM (SPM-9600, Japan) was utilized to measure the surface properties of the coatings, including morphology and roughness (Ra and Rz). Measurements were conducted in ambient air using an AFM scanner in tapping mode at a scanning rate of 0.7016 Hz and a scanning area of 10 μm × 10 µm. The coatings were measured in five different areas for statistical analysis. Fourier Transform Infrared (FTIR) spectroscopy and Raman spectroscopy were used to demonstrate the grafting of EDC onto SWNTs. The FTIR analysis was investigated using a Nicolet 6700 (Thermo Nicolet Corp., Madison, WI, United States). An 532 nm Nd:YAG laser was used as the excitation source for the Raman spectra collected with a LabRAM HR (HORIBA Jobin Yvon SAS, Longjumeau, France).

### 2.4 Isolation and Cultivation of BMSCs

Rat BMSCs were isolated from the bone marrow of the femurs and tibias of four-week-old Sprague-Dawley (SD) rats obtained from the State Key Laboratory of Oral Diseases, as described previously (Luo et al., 2017). All experiments were conducted following the ethical protocol approved by the Committee of Ethics of Sichuan University. An α-minimum essential medium (α-MEM) (Gibco, United States) was used to culture the cells along with 10% fetal bovine serum (Hyclon, United States) and 1% penicillin and streptomycin (Solarbio, China). The medium was changed every 3 days and incubated at 37°C with 5% humid CO_2_. Isolating BMSCs was achieved by removing nonadherent cells after 48 h of culture and maintaining adherent cells. After cells had reached 80%–90% confluence, they were passaged with 0.25% trypsin in 1 mM EDTA (Sigma).

### 2.5 Identification of BMSCs

Cells from the third generation were collected and digested using trypsin/EDTA. The reactions were stopped by adding serum-filled α-MEM culture media. A 1.5 × 10^6^ cell/ml suspension was prepared with phosphate-buffered saline (PBS) and separately removed into ten expanded polyethylene tubes. The phenotype of isolated cells was determined by analyzing the antigens on their surfaces. In brief, 10 µl of fluorescein isothiocyanate (FITC) or phycoerythrin (PE): FITC anti-rat CD11b/c, FITC anti-rat CD44H, PE anti-rat CD54, and PE anti-rat CD90 (Wuhan Boster Biological, Wuhan, Hubei, China) were added to both the tubes and the homotype control groups. One tube of unstained cells was prepared as a control for the antibodies. After mixing, the solutions were placed in the dark for 30 min at 4°C. Following this, 3 ml of PBS was added to each tube, mixed, and centrifuged for 5 min at 1000 rpm. The supernatants were discarded. This process was repeated several times. The expression levels of the cell-surface antigens CD11b/c, CD44H, CD54, and CD90 were measured by flow cytometry (Epics XL, Beckman Coulter, FL, United States).

### 2.6 *In Vitro* Induction of BMSCs Differentiation

The prepared SWNTs/EDC composite suspensions (2 ml) were added to 6-well plates and air-dried under ultraviolet-ozone (UVO) ambient conditions at room temperature for 3 d. Afterward, the specimens were exposed to UV light for 30 min, flushed with PBS twice, and then used for the following induction. Purified third-generation BMSCs were cultured on the surface of different ratios of SWNTs/EDC composites to induce the differentiation of BMSCs into VEC-like cells. After 7 d of culture, the BMSC-VEC morphology was studied, and images were recorded.

### 2.7 Cell Proliferation

After 1, 3, 5, and 7 days of culture, these cells were fixed and dehydrated to explore proliferation and morphology. The cell viability was quantitatively evaluated by Cell Counting Kit-8 (CCK-8, Dojindo, Japan) following the manufacturer’s instructions. Specifically, 100 µL of cell suspension was added to a 96-well plate with 10 µL of CCK-8 reagent. Then, the cell suspension was incubated for 2 h at 37°C. Optical density was determined by measuring the absorbance with a microplate reader (Bio-RAD iMark Microplate Reader, Hercules, CA, United States) at 450 nm. Each experiment was carried out in five replicates.

### 2.8 DiI-Ac-LDL Uptake and FITC-UEA-1 Binding Assay

To initially determine whether SWNTs/EDC composite could induce BMSCs to differentiate into VEC-like cells or not, adherent cells were stained with DiI-ac-LDL and FITC-UEA-1 (Sigma, United States) double fluorescence staining. Briefly, the cells grown on composites were cultured for 4 h at 37°C in a serum-free medium containing 10 mg/ml DiI-Ac-LDL. The cultures were washed three times in PBS, fixed for 20 min with 4% paraformaldehyde, and then stained with 10 mg/ml FITC-UEA-1 for 1 h. DiI-Ac-LDL incorporation and FITC-UEA-1 binding were detected by confocal microscopy (Leica Microsystems GmbH). DiI-Ac-LDL and UEA-1 dual-stained cells were identified as VEC-like cells.

### 2.9 Real-Time Polymerase Chain Reaction

The marker gene expression of VEC-like cells was analyzed by real-time PCR (RT-PCR). After BMSCs were cultured on SWNTs and SWNTs/EDC composites for 14 days, the total RNA from the samples was extracted using an RNeasy Mini kit (Qiagen, Valencia, California). For complementary DNA (cDNA), mRNA was reverse transcribed into cDNA with PrimeScript^®^ RT Master Mix (Takara Bio, Japan) following the manufacturer’s instructions. This study utilized a Fast Start Universal SYBR Green Master Mix (Roche) and a CFX Connect Real-Time PCR Detection System (Bio-Rad, Hercules, CA, United States) to perform quantitative RT-PCR. The sequences of primers for CD31, vWF, VEGF, and VEGF-R2 genes are given in Table 1. The samples were initially exposed to 95°C for 10 min, followed by 45 cycles at 95°C for 1 min and 60°C for 1 min. All reactions were carried out in a 10 μL reaction volume. The gene expression levels were calculated using the comparative 2^−ΔΔCT^ method, and β-actin was used as endogenous control. Each specimen was analyzed in triplicate.

**TABLE 1:** The primers and probes for real-time PCR.

Target	Forward primer (5′ to 3′)	Reverse primer (5′ to 3′)
vWF	ACC​TTG​GTC​ACA​TCT​TCA​CAT​TCA​CTC	AAG​TCA​TTG​GCT​CCG​TTC​TCA​TCA​C
CD31	TGT​CAA​GTA​AGG​TGG​TGG​AGT​CT	AGG​CGT​GGT​TGG​CTC​TGT​T
VEGF	AGG​GAA​GAG​GAG​GAG​ATG​AG	GCTGGGTTTGTCGGTGTT
VEGF-R2	CTG​GCT​ACT​TCT​TGT​CAT​CAT​CCT​ACG	TGG​CAT​CAT​AAG​GCA​GTC​GTT​CAC

vWF, von Willebrand Factor; CD31, platelet endothelial cell adhesion molecule-1; VEGF, vascular endothelial growth factor; VEGF-R2, vascular endothelial growth factor receptor 2.

### 2.10 Statistical Analysis

All experiments were conducted in triplicate, and all results are expressed as means ± standard deviation. We performed statistical analysis independently using a Student’s t-test and one-way analysis of variance (ANOVA) in conjunction with a Student-Newman-Keuls multiple comparison test (SNK). The differences were considered statistically significant at *p <* 0.05 and very significant at *p* < 0.01, respectively.

## 3 Results and Discussion

**FIGURE 1:**
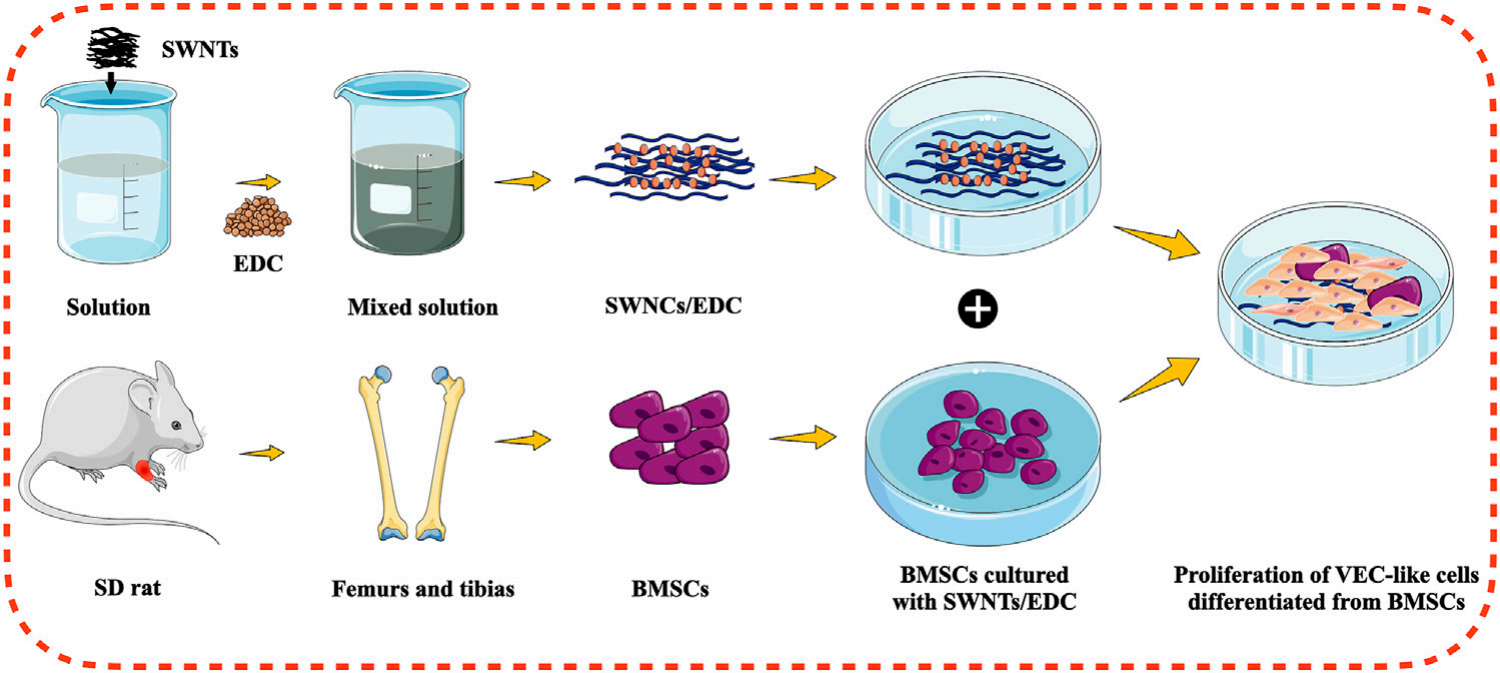
Schematic illustration for the differentiation of BMSCs into VEC-like cells using functionalized SWNTs.

Tissue engineering involves combining cells, scaffolds, and signaling molecules to restore damaged organs to their original shape and function. Unfortunately, a lack of adequate blood supply limits tissue engineering’s clinical application (Shiekh et al., 2018). Hence, the fabrication of an adequate bioactive scaffold for bone tissue engineering is considered essential to stimulate angiogenesis. VECs are essential aspects of vascularization in bone regeneration. Studies have shown that BMSCs can differentiate into various cells, including VECs, depending on the microenvironment containing growth factors, scaffold formulation, and other culture conditions (Chen et al., 2018). CNTs are a promising bioactive scaffold for bone tissue engineering because of their outstanding properties. Several researchers have reported the *in vitro* and *in vivo* bone regeneration effectiveness of CNTs. Furthermore, CNTs-reinforced composites can increase BMSC proliferation and spread and enhance mRNA and protein expressions of bone sialoprotein and osteocalcin (Arumugam and Ju, 2021). However, the effect of CNTs on vascularization is still minimal. In the current study, we induced the BMSCs to differentiate into VEC-like cells *in vitro* by SWNTs/EDC to assess the potential of CNTs to induce angiogenesis, as schematically illustrated in Figure 1.

### 3.1 Physicochemical Characterization of SWNTs/EDC Composites

#### 3.1.1 Purification and Morphology of SWNTs

**FIGURE 2:**
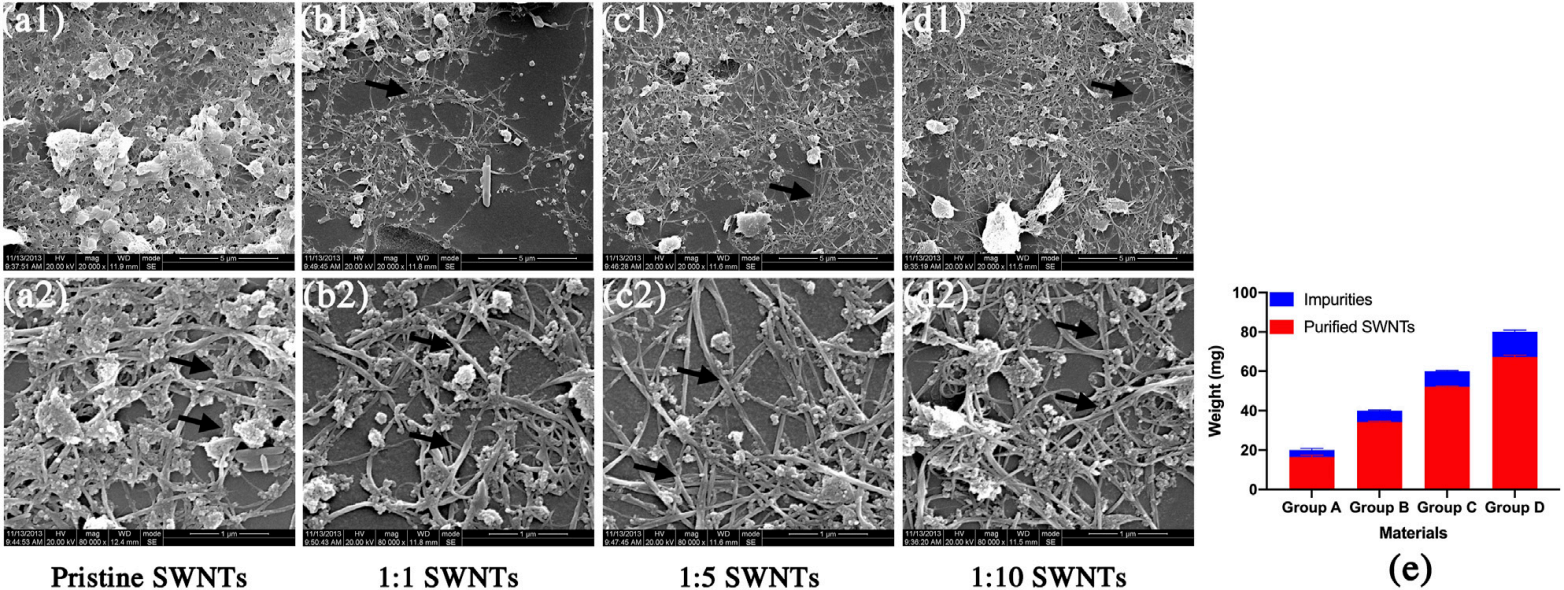
Scanning electron micrographs and weight loss of pristine SWNTs and purified SWNTs. **(A)** pristine SWNTs, **(B)** 1:1 SWNTs, **(C)** 1:5 SWNTs; **(D)** 1:10 SWNTs; Scale marker: 5 μm; 1 μm; Magnification: 1: 20,000; 1: 80,000; **(E)** Weight loss of SWNTs after purification by sintering.

This work used SWNTs with a diameter of 1–2 nm and a length of 5–30 μm, as shown in Figure 2. The pristine SWNTs in this study are entangled and aggregated due to attracting van der Waals forces (Huang et al., 2021). Similar to previous reports (Im et al., 2017), the pristine SWNTs contain many impurities (Figures 2A1,A2), including catalysts and amorphous carbon. These impurities may be introduced in the manufacturing process and contribute to the agglomeration of SWNTs. The pristine SWNTs are purified by a high-temperature sintering method to improve their dispersion and surface compositions. Figures 2B1–D2 shows that the purified SWNTs contain fewer amorphous carbon particles on their surface and are distributed separately. After purification, 17.68%, 14.19%, 13.21%, and 15.94% of impurities are removed (Figure 2E), indicating that the pristine SWMTs have been purified, consistent with the results of the SEM (Figures 2A–D–D).

#### 3.1.2 Morphology and Dispersion of SWNTs/EDC Composites

**FIGURE 3:**
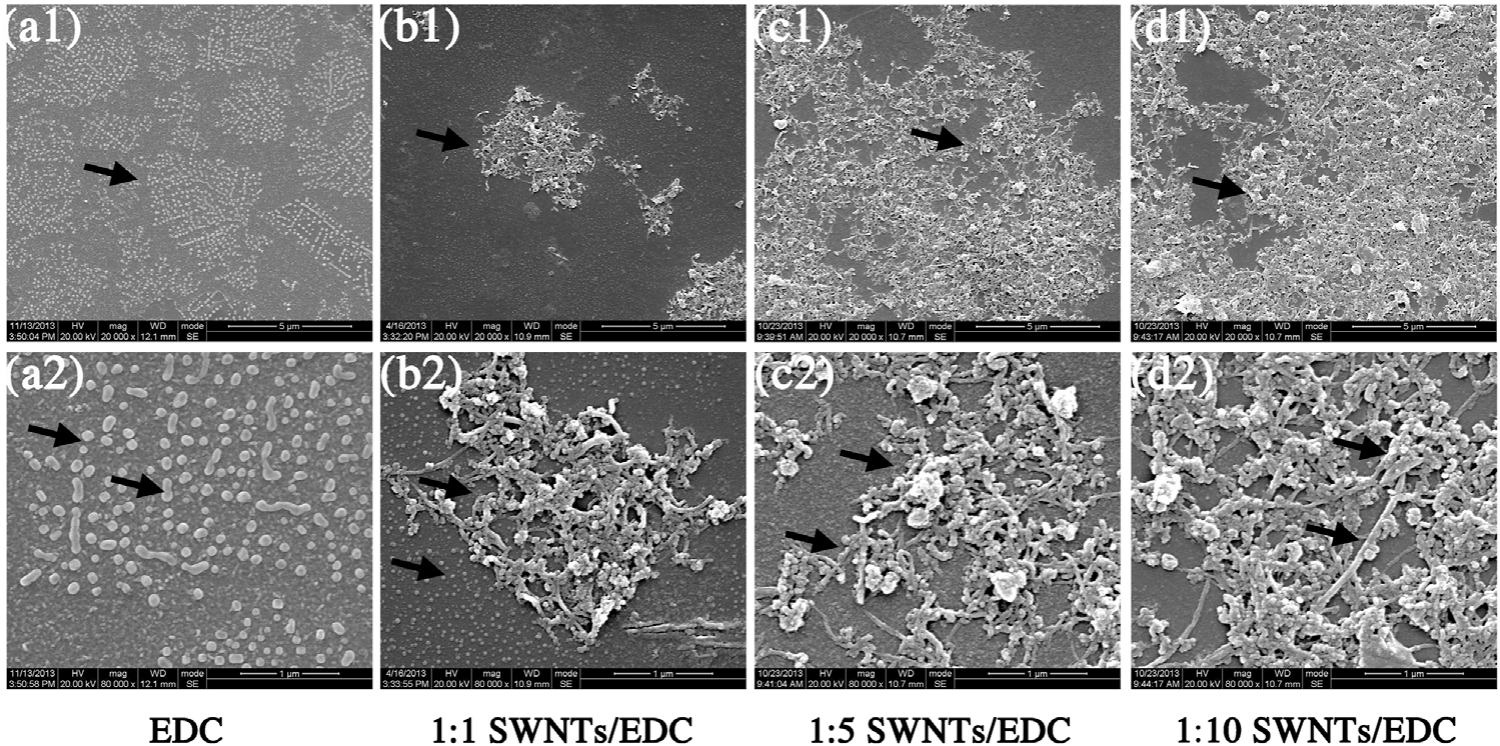
Scanning electron micrographs of EDC, and different ratios of SWNTs/EDC composites. EDC has an elliptical or irregular granule shape, and the SWNTs treated with stirring, ultrasonication and centrifugation are distributed separately. EDC is embedded in the SWNTs matrix and appears uniformly distributed throughout the composites. **(A)** EDC, **(B)** 1:1 SWNTs/EDC, **(C)** 1:5 SWNTs/EDC; and **(D)** 1:10 SWNTs/EDC; Scale marker: 5 μm; 1 μm; Magnification: 1: 20,000; 1: 80,000.

The SWNTs/EDC suspensions (1:1, 1:5, and 1:10) are fabricated by the solvent evaporation technique. Ultrasonication and centrifugation techniques are used to break the agglomeration of SWNTs and improve the dispersion of SWNTs/EDC suspensions, which is essential for the mechanical properties and bioactivity of the composites. The SEM results show that EDC has an elliptical or irregular granule shape (Figures 3A1,A2) embedded in the SWNTs matrix (Figures 3B1–D2). In addition, EDC appears uniformly distributed throughout the composites, attributed to phase separation during the ultrasonication and centrifugation processes (Manilov et al., 2019). Additionally, the numbers of SWNTs and EDC were increased with an increase in the SWNTs concentration.

#### 3.1.3 Atomic Force Microscopy

**FIGURE 4:**
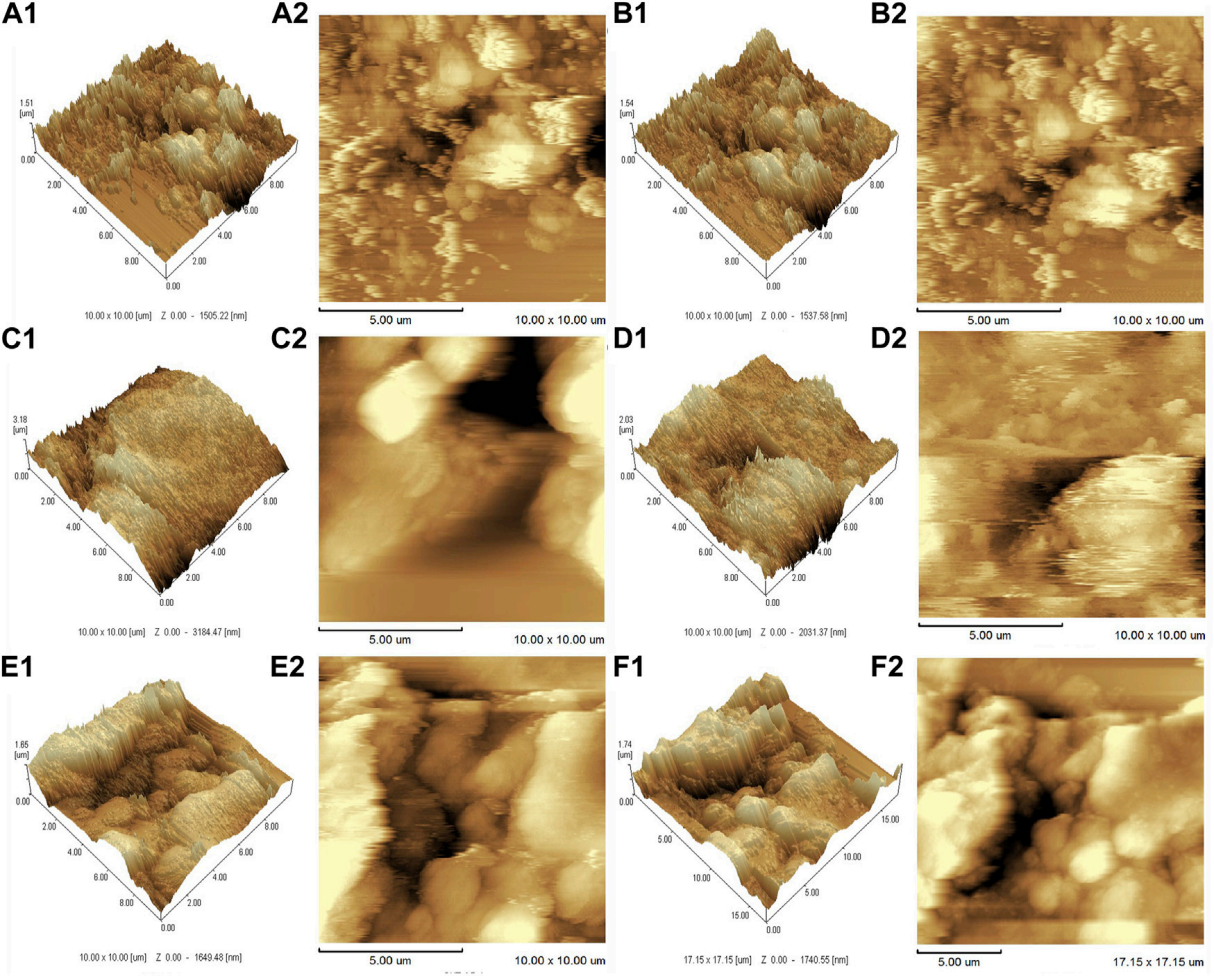
AFM analysis of different ratios of SWNTs and SWNTs/EDC. 3D AFM topographic micrographs. **(A1)**, 1:1 SWNTs; **(B1)**, 1:1 SWNTs/EDC; **(C1)**, 1:5 SWNTs; **(D1)**, 1:5 SWNTs/EDC; **(E1)**, 1:10 SWNTs; and **(F1)**, 1:10 SWNTs/EDC. 2D AFM topographic micrographs. **(A2)**, 1:1 SWNTs; **(B2)**, 1:1 SWNTs/EDC; **(C2)**, 1:5 SWNTs; **(D2)**, 1:5 SWNTs/EDC; **(E2)**, 1:10 SWNTs; and **(F2)**, 1:10 SWNTs/EDC.

**FIGURE 5:**
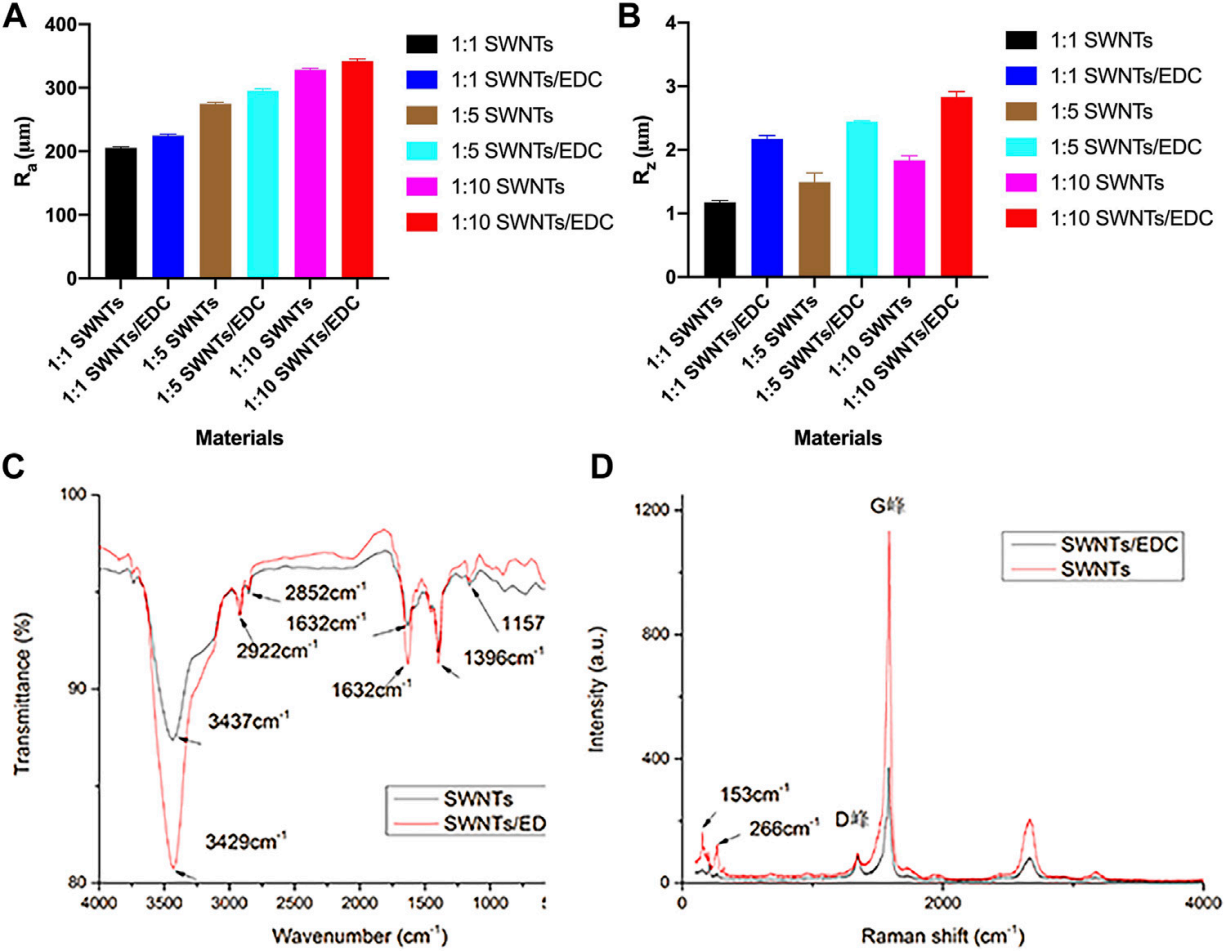
Physiochemical characterization of SWNTs and SWNTs/EDC. Surface roughness: **(A)** R_a_; **(B)** R_z_. The arithmetic average roughness (R_a_) of the SWNTs (1:10) and SWNTs/EDC (1:10) are 328.544 ± 2.443 and 341.707 ± 3.795, and the ten-point average roughness (R_z_) of the SWNTs (1:10) and SWNTs/EDC (1:10) are 1.834 ± 0.071 and 2.833 ± 0.083, respectively. **(C)** FTIR spectra of the pristine SWNTs and SWNTs/EDC composite; **(D)** Raman spectra of the pristine SWNTs and SWNTs/EDC composite.

The AFM results (Figure 4) further demonstrated that the SWNTs were closely attached and uniformly distributed, further demonstrating EDC’s successful grafting on SWNTs. In addition, because the diameter of the added SWNTs was 1–2 nm, the surface topography of the composites was assumed to be nanoscale (Kim et al., 2016). As reported, nanostructured surfaces can modulate cell and tissue functions significantly, and that surface modification on a nanoscale can enhance bone regeneration (Rasouli et al., 2018). AFM also estimated the surface roughness of SWNTs/EDC composites. The arithmetic average roughness (Ra) (Figure 5A) of the SWNTs (1:10) and SWNTs/EDC (1:10) are 328.544 ± 2.443 and 341.707 ± 3.795, and the ten-point average roughness (Rz) of the SWNTs (1:10) and SWNTs/EDC (1:10) (Figure 5B) are 1.834 ± 0.071 and 2.833 ± 0.083, respectively. As reported, both the chemical characteristics and surface topography of materials play an essential role in their biocompatibility and bioactivity (Al-Jumaili et al., 2017). Therefore, it is assumed that the surface topography of SWNTs/EDC composites might positively affect cell function.

#### 3.1.4 Fourier Transform Infrared Spectroscopy

Figure 5C shows the recorded FTIR spectra of pristine and functionalized SWNTs. The pristine SWNTs spectra show infrared absorptions at 2922 and 2852 cm^−1^ are related to -CH stretching vibrations and the typical carbonyl (C=O) stretch, respectively. Additionally, bands at 1157 cm^−1^ related to C–O tension are observed. Noteworthy is that even though SWNT structures lack hydroxyl groups, a small signal is observed at 3437 cm^−1^, which can be explained by OH stretching tension induced by moisture adsorbed at the interface. After treatment with EDC, the SWNT spectra show peaks at 3429 and 1632 cm^−1^, which correspond to the stretching vibrations of hydroxyl groups (-NH) and inplane bending vibrations (-NH) of EDC adsorbates, respectively (Kumar et al., 2015). Hence, the presence of these peaks indicates the formation of SWNTs/EDC composite coatings on the slide surface.

#### 3.1.5 Raman Spectroscopy

Furthermore, Figure 5D shows the recorded Raman spectrum of pristine and functionalized SWNTs. The purity of the SWNTs can be determined by the shape of the G-band and D-band and from the relative area intensities of the G-band to D-band (i.e., IG/ID). The higher the ratio is, the higher the purity of the SWNTs (Huang et al., 2017). Herein, the IG/ID values of SWNTs and SWNTs/EDC are 11.7 and 4.2, respectively, demonstrating the presence of EDC. Additionally, the diameters of the SWNTs were calculated from the radial breathing modes (RBMs) (Kröckel et al., 2020). In order to analyze Raman spectra, the formula ωRBM = A/d + B is used, where A = 234 cm^−1^, B = 10 cm^−1^, ωRBM is the radial breathing mode, and d is the diameter of the nanotube. In this study, the diameters of the SWNTs and SWNTs/EDC are 1.6 and 0.9 mm, respectively. The difference in diameters between SWNTs and SWNTs/EDC can further demonstrate that EDC is successfully grafted. As reported, the diameter distribution of SWNTs is expected to be affected by the change in the bundle diameter but is unaffected by purification (Ramesh et al., 2006). Therefore, the diameter change may be attributed to the ultrasonication and centrifugation processes instead of high-temperature vacuum sintering, which improves the dispersion of SWNTs/EDC suspensions. In addition, because the diameters of the SWNTs and SWNTs/EDC are 1.6 and 0.9 mm, the surface topography of the materials can be regarded as nanoscale. It was demonstrated that nanostructured scaffold surfaces positively affected tissue engineering (Wang et al., 2018). Thus, we could assume that the morphology of SWNTs and SWNTs/EDC composites contributes to cell adhesion and proliferation.

### 3.2 Effects of SWNTs/EDC on the Differentiation of BMSCs Into VEC-Like Cells

#### 3.2.1 Morphological Features and Phenotypic Characterization of BMSCs

**FIGURE 6:**
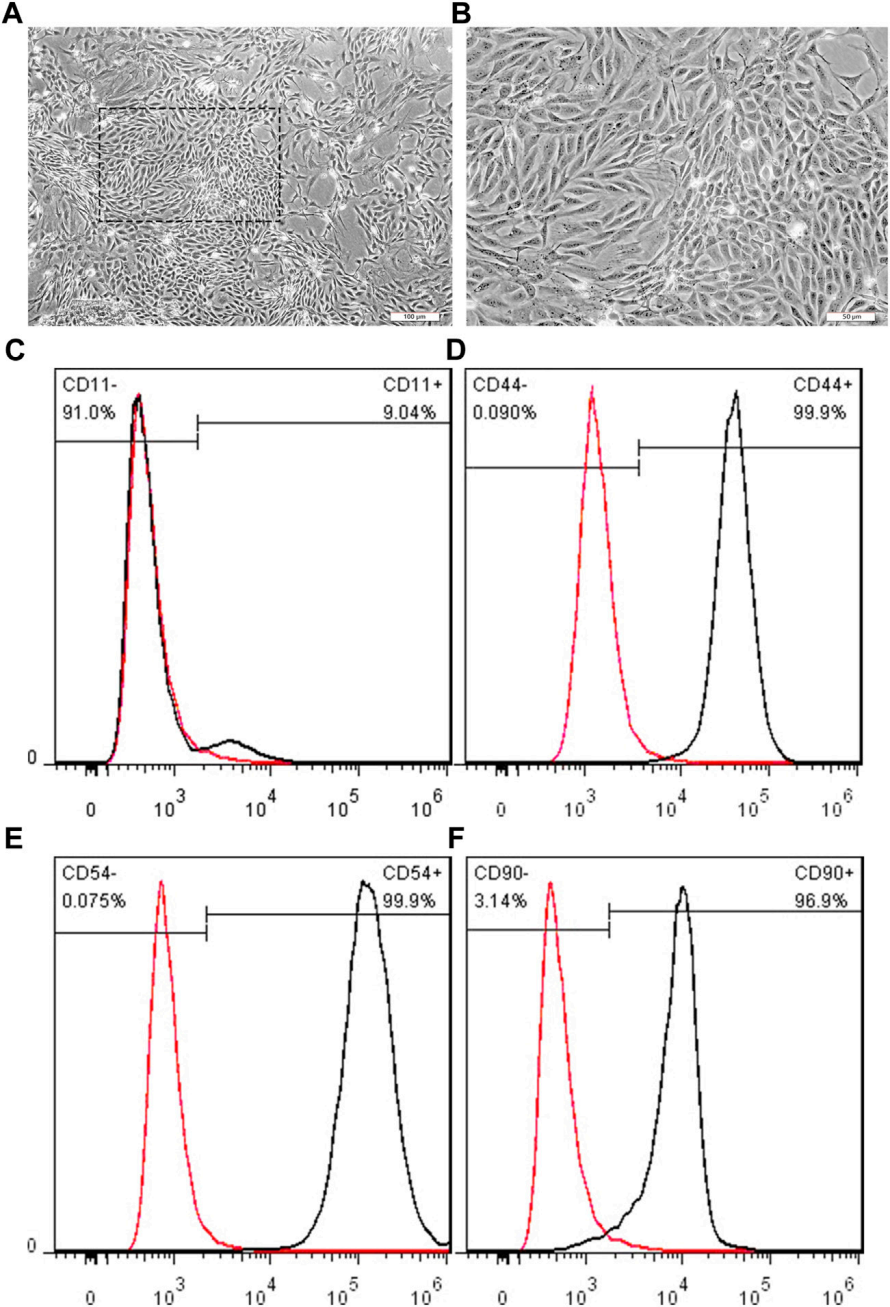
Representative images showing the BMSCs morphologies at three passages. **(A, B)** BMSCs display clearly bonded long, spindle shapes, with full central nuclei. Scale bar: 100 μm **(A)**, 50 μm **(B)**; Cell surface antigen CD11b/c **(C)**, CD44 **(D)**, CD54 **(E)**, and CD90 **(F)** was detected by flow cytometry, respectively. Each antigen positive cells were gated (black peak), compared to the negative control (red peak).

To investigate the effect of EDC-modified SWNTs on vascularization, BMSCs were obtained from rat bone marrow and then verified. Purified BMSCs are prepared after digesting and passage of adherent-screened cells. After the passage to the third generation, we obtained purified BMSCs. As shown in Figures 6A,B, BMSCs are long and spindle-shaped, have full central nuclei, and are arranged radially or spirally, which is consistent with previous reports (Wan et al., 2012). Furthermore, the lineage-specific markers were detected using flow cytometry to characterize the phenotype of isolated cells. In this study, the cells expressed high levels of CD 44 (99.9%, Figure 6D), CD54 (99.9%, Figure 6E) and CD90 (96.9%, Figure 6F), while low levels of CD11b/c (9.04%, Figure 6C). These data reveal that the BMSCs used in the present study were mesenchymal type and exhibit typical BMSCs characteristics (Gugjoo et al., 2019).

#### 3.2.2 VEC-Like Cells Differentiation of BMSCs

**FIGURE 7:**
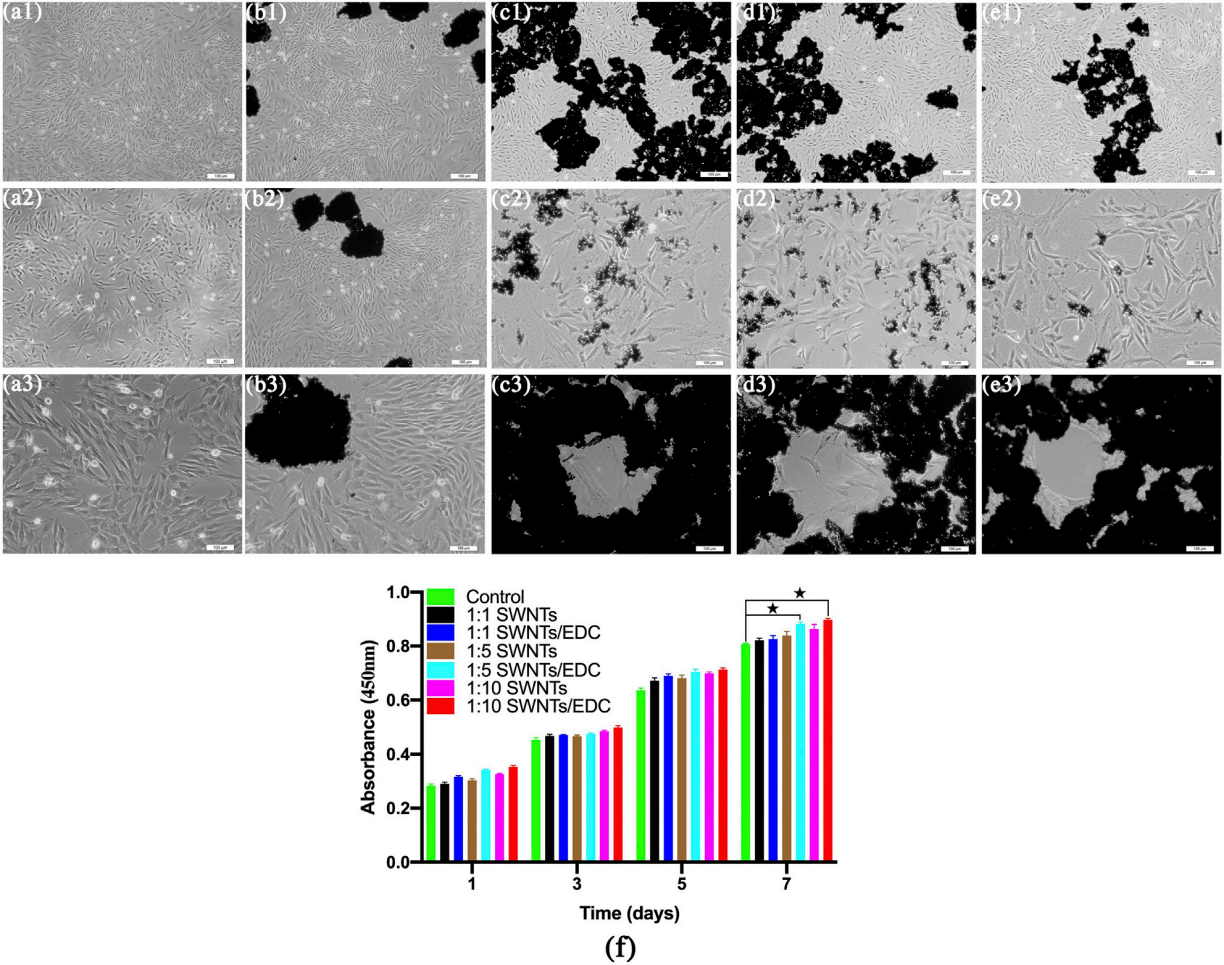
After 7 d of culture, the morphology and proliferation of BMSC-VEC were studied. BMSCs cultured with 1:10 SWNTs/EDC show a capillary-like appearance, whereas cells in the control group show only a dispersed pattern. Control **(A1–A3)**; SWNTs, **(B1–B3)**; 1:1 SWNTs/EDC, **(C1–C3)**; 1:5 SWNTs/EDC, **(D1–D3)**; and 1:10 SWNTs/EDC, **(E1–E3)**. **(F)** CCK-8 assays for the proliferation of BMSCs on the standard plate, SWNTs, and SWNTs/EDC surfaces after 1, 3, 5, and 7 days *in vitro* culture. Data represents mean ± SD from five cultures. Significant differences: *p* < 0.05.

SWNTs/EDC composites were evaluated for their effects on cell morphology and viability by seeding BMSCs onto standard plates, SWNTs, and SWNTs/EDC surfaces for 1 d, 3 d, 5 d, and 7 d of incubation. BMSCs cultured on the standard plate were served as the control group. The cell morphology and viability are shown in Figure 7. The shape, morphology, and growth rate of BMSCs grown on SWNTs and SWNTs/EDC at 1 and 3 d are similar to those cultured on a standard culture plate. With time prolongation, the number of BMSCs grew on the standard plate, SWNTs, and SWNTs/EDC surfaces significantly increased, and few dead cells were observed, suggesting that the BMSCs are viable and the prepared SWNTs, and SWNTs/EDC composites have good biocompatibility. Furthermore, compared with cells grown on the standard plate, BMSCs grown on SWNTs and SWNTs/EDC composites showed more wide-spreading morphology, indicating that the SWNTs and SWNTs/EDC composites could enhance the cell adhesion and growth. Furthermore, Figure 7E shows that BMSCs cultured with 1:10 SWNTs/EDC show a capillary-like appearance, but control cells show only a scattered pattern (Figures 7A,B). This result may be associated with the superior biocompatibility and rough surface topography of SWNTs and SWNTs/EDC composites (Qin et al., 2022).

#### 3.2.3 Proliferation of VEC-Like Cells Differentiated From BMSCs

The adhesion and proliferation rates of BMSCs on the standard plate, SWNTs, and SWNTs/EDC surfaces were studied by CCK-8 assays for 1, 3, 5, and 7 days. As shown in Figure 7F, the optical density (OD) values for all groups increased with time prolongation. Besides, higher adhesion and proliferation rates are observed for BMSCs cultured on both the SWNTs and SWNTs/EDC surfaces compared to cells grown on the plate. These results demonstrate the excellent biocompatibility and bioactivity of SWNTs and SWNTs/EDC and demonstrate their potential to serve as growth media for BMSCs without being toxic. The OD values for SWNTs/EDC composites are more significant than for the standard plate group might be attributed to the functionalization of SWNTs and EDC, which facilitated cell growth and proliferation. After incubation for 7 days, the adhesion and proliferation rates of 1:10 SWNTs/EDC are better than those of the other groups (*p* < 0.05), which means the composite at mass/volume ratios 1:10 had the best enhancement of proliferation and differentiation of BMSCs. This phenomenon is attributed to the nanopores and roughness of SWNTs/EDC surfaces, in which BMSCs can proliferate and differentiate in a three-dimensional environment (Minagar et al., 2015). Furthermore, CNTs have a nanofibrous structure that makes them ideal as biomimetic analogs for extracellular matrix proteins (ECM), specifically collagen (Jing et al., 2017). The CNTs might act similarly to collagen fibers in the formation of bone matrix, regulating crystal nucleation and the growth of the inorganic component. Above all, CNTs in scaffolds for bone regeneration could be a unique additive.

#### 3.2.4 FITC-UEA-1 and DiI-Ac-LDL Double Staining

**FIGURE 8:**
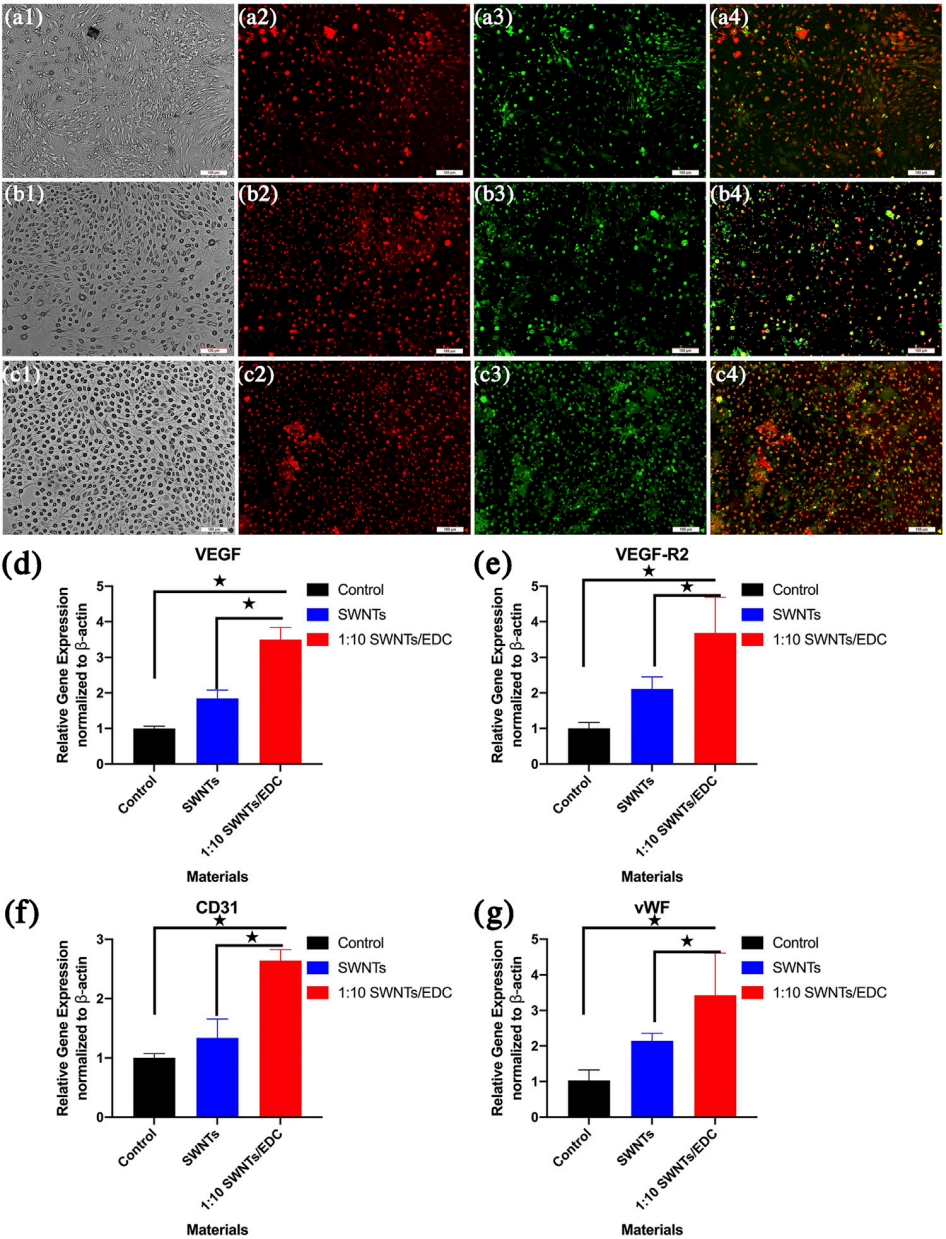
Dil-Ac-LDL uptake, FITC-UEA binding and merged image of BMSC-VECs were determined by Fluorescence Microscope. Differentiated BMSC-VECs are positive for both the uptake of DiI-ac-LDL (red) and binding of FITC-UEA-1 (green). Double-positive cells appearing yellow were identified as differentiating VEC-like cells. **(A)** 1:1 SWNTs/EDC; **(B)** 1:5 SWNTs/EDC; and **(C)** 1:10 SWNTs/EDC. The RNA expression of representative endothelial cell markers **(D)** VEGF, **(E)** VEGF-R2, **(F)** CD31, and **(G)** vWF in BMSCs grown on SWNTs and the SWNTs/EDC composites were determined by RT-PCR. The VEGF-R2 expression level of SWNTs/EDC is significantly higher than that of SWNTs and the control group. Significant differences: *p* < 0.05.

The scaffolds can promote the formation of vasculature and angiogenesis *in vivo*, providing nutrients and oxygen to the cells after implantation, which is vital for bone tissue regeneration (Gholobova et al., 2020). SWNTs/EDC composite was used to induce BMSCs to differentiate into VEC-like cells. The phenotype of BMSC-VECs was identified by applying immunofluorescent staining with FITC-UEA-1 and DiI-Ac-LDL double staining (Figures 8A–C–C). In differentiated BMSC-VECs, endocytotic vesicles were fluorescent red due to the uptake of DiI-ac-LDL within the cytoplasm. Simultaneously, the cell membrane appeared green after staining with FITC-UEA-1. The overlay (yellow) shows that all acLDL-positive cells are also UEA-1-positive (Huang et al., 2021). In contrast, undifferentiated BMSCs do not bind UEA-1 or ac-LDL. In this study, around 78.3% ± 4.2% of adherent cells exhibit dual positivity for 1:10 SWNTs/EDC composites (Figure 8C4), suggesting that they express both ac-LDL scavenger receptors and UEA-1 ligands. The uptake of DiI-Ac-LDL and the ability to bind lectin have previously been considered biomarkers of differentiating VECs (Li et al., 2019). BMSC-VECs are mononuclear bone marrow cells with partial endothelial function and their ability to differentiate, and BMSC-VECs can bind to UEA-1 and ac-LDL (Liu et al., 2021).

#### 3.2.5 The RNA Expression of Representative Endothelial Cell Markers

However, some studies have found that binding to UEA-1 and ac-LDL uptake, previously used to identify differentiating VECs, are not specific for VECs. Thus, to further demonstrate that VEC-like cells successfully differentiated from BMSCs under the induction of SWNTs/EDC composite, the RNA expression of representative endothelial cell markers VEGF, VEGF-R2, CD31, and vWF were determined by RT-PCR (Figures 8D–G–G). As previously reported, VEGF/VEGFR confers the ability for stem cells to migrate and differentiate into differentiated endothelial cells that are correctly positioned and differentiated (Chen et al., 2020). RT-PCR results revealed that the expression of the endothelial cell markers, VEGF, and VEGF-R2, was significantly higher in the SWNTs/EDC composite group than that in the control group and SWNTs group (*p* < 0.05) (Figures 8D,E). VEGF is a particular factor that stimulates the growth of endothelial cells (Cao et al., 2021). VEGF facilitates angiogenesis by increasing the permeability of the vascular wall, decreasing its extracellular matrix content, and enhancing the migration and proliferation of vessel endothelial cells. Moreover, VEGFR-2 is considered one of the earliest markers for endothelial and hematopoietic cells (Ribatti et al., 2020). These findings suggest a successful differentiation of BMSCs to VEC-like cells.

To further understand that BMSCs are differentiated into VEC-like cells by SWNTs/EDC composite, we investigated the expression of CD31 and vWF. The immunoglobulin protein CD31 is found at the intercellular junctions of VECs, which play an essential role in the angiogenesis process and new bold vessel formation (Duan et al., 2015). The protein vWF is produced by endothelial cells and has been used for years as a marker for distinguishing differentiated endothelial cells (Mojzisch and Brehm, 2021). The expression of vWF in endothelial cells cultured *in vitro* is upregulated by angiogenic factors like VEGF. As shown in Figures 8F,G, SWNTs and SWNTs/EDC composite groups showed significantly higher expression of endothelial cell markers CD31 and vWF compared to the control group (*p* < 0.05). These data confirmed that the SWNTs/EDC composite could induce endothelial-like cell differentiation of BMSCs.

The *in vitro* data indicates that SWNTs/EDC exhibits more potential for angiogenesis than SWNTs. These results demonstrate that SWNTs/EDC can successfully induce BMSCs differentiation into VEC-like cells, explained by the following reasons. First, BMSCs are non-hematopoietic multipotent stem cells and have an intrinsic ability to differentiate into functional cell types, including VECs (Nammian et al., 2021). Second, SWNTs are excellent substrates for cellular growth, and the rough surface of SWNTs/EDC is good for cell proliferation and differentiation (Al-Jumaili al., 2017; Wang et al., 2018). More importantly, SWNTs are in tubular structures suitable for the differentiated formation of VEC-like cells from BMSCs (Cheng et al., 2020). For example, SWNTs share characteristics with a natural extracellular matrix (ECM), which is ideal for scaffolds to promote bone formation (Arumugam and Ju, 2021). Further, SWNTs should have a high degree of flexibility and elasticity, facilitating cell attachment (Shadjou and Hasanzadeh, 2016). Another critical common feature of SWNTs and ECM is porosities of similar diameters (Tonelli et al., 2012). Due to their high porosity, nanotubes are good candidates for scaffolding because the natural ECM is highly porous, which is essential for integrating tissues. These preliminary results confirm the efficiency and efficacy of the SWNTs/EDC composite in inducing the differentiation of VEC-like cells from BMSCs.

The results support the hypothesis that SWNTs/EDC composites can promote BMSCs differentiation into VEC-like cells and positively affect angiogenesis and bone regeneration. Interestingly, our findings in this study may provide better insight into how SWNTs influence vascularization and osteoinduction during bone regeneration. One limitation of our study is the inability to confirm endothelial cell migration, tube formation, and angiogenesis potential. Therefore, our future studies will evaluate whether SWNTs/EDC composite has angiogenic potential to form mature tubular microvessels *in vivo*. In addition, we can further study whether SWNTs/EDC composite can advance the formation of vascularized constructs for bone regeneration.

## 4 Conclusion

In this study, SWNTs were purified to improve their dispersion status and surface compositions, and SWNTs/EDC composite was successfully prepared. The FTIR spectra and Raman spectra results confirmed the formation of SWNTs/EDC composites. The AFM results demonstrated that the SWNTs were closely attached and uniformly distributed. The surface topography of the SWNTs/EDC composites presents a rough surface, which may positively affect cell function. *In vitro* cell culture revealed that SWNTs and SWNTs/EDC composites exhibited excellent biocompatibility and bioactivity, and SWNTs/EDC composites at mass/volume ratios 1:10 had the best enhancement of proliferation and differentiation of BMSCs. Moreover, after culture with SWNTs/EDC composite, approximately 78.3% ± 4.2% of total adherent cells are dual-positive for FITC-UEA-1 and DiI-Ac-LDL double staining. Additionally, the RNA expression of representative endothelial cell markers VEGF, VEGF-R2, CD31, and vWF was significantly higher in the SWNTs/EDC composite group than in the control and SWNTs group. Although the active mechanism of SWNTs/EDC composited on BMSCs differentiation remains unclear, the results suggested that SWNTs/EDC composite can promote BMSCs differentiation into VEC-like cells and positively affect angiogenesis and bone regeneration.

## Data Availability

The original contributions presented in the study are included in the article/supplementary material, further inquiries can be directed to the corresponding authors.
